# Sharing digital health data responsibly: Balancing open science with participant privacy

**DOI:** 10.1371/journal.pdig.0001377

**Published:** 2026-04-24

**Authors:** Camille Nebeker, Shahin Samiei, Santosh Kumar

**Affiliations:** 1 Herbert Wertheim School of Public Health and Human Longevity Science, University of California San Diego, La Jolla, California, United States of America; 2 Department of Computer Science, University of Memphis, Memphis, Tennessee, United States of America; Instituto Politécnico Nacional Escuela Superior de Medicina: Instituto Politecnico Nacional Escuela Superior de Medicina, MEXICO

## Abstract

Data sharing is essential for modern science, advancing transparency, reproducibility, and discovery. Emerging evidence shows that certain digital health data, particularly high-frequency accelerometry from body-worn sensors, carry re-identification risks even when de-identified. In 2023, the National Institutes of Health published its Data Management and Sharing (DMS) Policy formalizing a commitment to openness by requiring all funded researchers to share scientific data. However, the policy did not anticipate that raw motion signals collected by wearable devices can function as biometric identifiers. The WristPrint study demonstrated that a single day of raw accelerometry data could be used to re-identify individuals with 96% accuracy. Related research in gait detection shows that as few as ten steps may be enough to uniquely identify someone. These findings highlight gaps in current data sharing policies and the need for tailored guidance. We argue that policy updates, enforceable data use agreements, and educational initiatives are essential to align openness with protection. The path forward is not to retreat from data sharing but to share more wisely, safeguarding participant trust while sustaining scientific progress.

## Author summary

Data sharing fuels scientific discovery, but new evidence shows that certain types of digital health data (i.e., raw motion signals from wearable sensors) can reveal more about individuals than expected. Research now demonstrates that even a few steps of walking can uniquely identify someone, raising important privacy concerns. In this article, we argue for policies and education that both enable open science and safeguard research participants.

We provide a multidisciplinary perspective, spanning research ethics, data science, and computational health, on how to share data responsibly in the era of digital health.

The sharing of health data has become central to modern biomedical science. By enabling harmonization of datasets, large-scale pooling of samples, and new analytic approaches, data sharing has transformed our ability to study human health. The U.S. National Institutes of Health (NIH) underscored this commitment in its 2023 Data Management and Sharing (DMS) Policy, which requires all NIH-funded researchers to submit DMS plans [1].

The benefits of sharing health data are clear. Large initiatives such as the UK Biobank, the National Health and Nutrition Examination Survey (NHANES), the Women’s Health Initiative, and the Baltimore Longitudinal Study of Aging provide researchers worldwide with access to granular physical activity and other health data [2–5]. These resources have enabled important discoveries in epidemiology, aging, and preventive medicine, and they represent the value of public investments in digital health research.

While the benefits to human health are promising, digital health data also introduces unique privacy risks. Unlike traditional identifiers such as names or addresses, the rich and continuous signals captured by body-worn sensors act as biometric signatures. The WristPrint study demonstrated that one day of raw accelerometry data was sufficient to re-identify individuals with 96% accuracy, even after removal of direct identifiers [6]. The fact that anonymized or de-identified raw accelerometer data can be combined with other data to re-identify people was neither known nor acknowledged when the current NIH data sharing policies were drafted. This observation aligns with a broader ethical critique that traditional de-identification practices have become somewhat obsolete in the era of machine learning and AI, where almost any data can be re-identified once linked with external sources. As Meeder and Doerr argue, dated ethics review and governance structures often misjudge risk by focusing on the presence or absence of direct identifiers rather than the re-identification potential inherent in high-dimensional digital traces associated with wearable sensor data [7].

Evidence from related work strengthens and provides background to this concern. Many years of research have shown that even brief walking sequences can identify individuals. The IDNet system achieved near-perfect recognition accuracy with fewer than five gait cycles, equivalent to fewer than 10 steps [8]. Smartwatch accelerometer data can likewise authenticate users within 10 s of walking, requiring only a handful of steps [9]. Smartphone and wearable gait authentication studies consistently demonstrate that short motion segments, once thought innocuous, are sufficiently distinctive to serve as a biometric signature [10,11]. Research using wearable inertial measurement units (IMUs) confirmed high accuracy rates across contexts, underscoring gait robustness as a biometric [12]. These findings align with broader surveys of gait recognition, which consistently highlight walking patterns as a reliable biometric across sensing modalities, from wearables to video [13]. A systematic review further concluded that de-identifying wearable datasets is often insufficient to eliminate re-identification risks, underscoring accelerometry and gait as especially sensitive modalities [14]. While much research has been focused on IMU sensing with respect to gait, growing research explores re-identification risk from other activities such as exercise or sports. Biosignals from other wearable sensing modalities such as electrocardiogram, wrist photoplethysmography, electroencephalogram, and others are also worthy of continued research consideration. [14].

It is unsurprising that, after many years of research in the gait detection context [15], high-frequency wrist-worn sensor data can also be used to detect movement patterns that are re-identifiable to an individual. The WristPrint study underscored this across different activities that people engage in their natural life, including walking, exercise, other nonstationary activities, and stationary activities. This evidence suggests that the ability to identify individuals from their routine movements is not a speculative risk, but a current reality. The privacy risk implications are significant. If raw sensor data are used to infer personal behaviors or health status, participants may be exposed to reputational, financial, or legal harms. Just as importantly, if participants lose trust that researchers can adequately protect their privacy, willingness to contribute data may decline. The credibility of science rests on maintaining this trust. Current policy provides some protection, but there are gaps to address. The NIH DMS Policy permits restrictions when privacy concerns are present, but does not provide explicit guidance on how to assess or mitigate risks from high-frequency digital health data.

Empirical biobank consent studies consistently document persistent comprehension challenges around key privacy and secondary use concepts [16]. Likewise, a systematic review finds that consent practices frequently emphasize assurances of de-identification even as participants express ongoing concerns about downstream uses [17]. While informed consent is necessary, it is not sufficient. Public data and digital health research increasingly involve populations with variable data and technology literacy [18]. Even when the participant understands that re-identification is possible, they are powerless to control how data are used once shared. Furthermore, recent peer-reviewed guidance demonstrates that datasets described as “anonymized” may still be vulnerable to re-identification through linkage or other analytic techniques [19]. Adding to these challenges, most data use agreements lack processes for enforcing expectations that researchers should not attempt to re-identify participants. Enforcement mechanisms tend to be limited to funding consequences (e.g., terminating awards) and do not extend to those outside of NIH oversight [20]. The enforceability of agreements outside of the context of NIH oversight may extend to other program sponsors (federal or private sector) or within the context of existing statute (e.g., HIPAA, FERPA, state privacy laws, or international laws where applicable such as GDPR). Though outside of such a scope would require reactive detection of an agreement breach, litigation, arbitration, or some other mediation mechanism, and penalties such as agreement termination, financial penalties, and/or other legal liabilities that would occur within the scope of such an agreement having been breached [21].

## Steps to enhance protections

To protect research participants while preserving the benefits of open science, we present the following steps: 1- Funders should issue guidance specific to high-frequency biosensor data, including recommended thresholds for aggregation (e.g., both temporally and/or feature-level) depending on the nature of high-frequency data collected (such as step count or activity or postural state), or when aggregation is impracticable, consider requirements for controlled-access repositories; 2- Researchers should be required to include sufficient risk-mitigation strategies in their DMS plans commensurate with the risk profile of the high-frequency data (e.g., sensor type, sensor sampling rate, duration of data collection, and intensity of activities collected) used in their data collection protocols, such as on-device data aggregation, privacy-preserving transformations and technical strategies such as differential privacy, statistical noise introduction and other statistical methods such as k-anonymity, l-diversity, and t-closeness [22], and/or secure analysis environments; 3- Consent processes must be updated to disclose re-identification risks in clear and accessible formats; 4- Downstream users must be bound by enforceable agreements that prohibit re-identification attempts; and, 5- penalties for misuse should be communicated and enforced by institutions as well as funding agencies. Risks associated with high-frequency sensor data persist long after participants enroll. Therefore, stronger oversight, such as enforceable data use agreements, clearer restrictions on re-identification attempts and improved governance structures is necessary to protect participants from downstream harms that informed consent alone cannot mitigate. “Guidelines that convey best practices, and regulations that establish enforceable requirements, responsive to the rapidly evolving capacity of scientific discovery, can help researchers, funders, and society to better manage risks to individuals while simultaneously fostering innovation.” Education is equally important and intrinsically complimentary to guidance and policy measures, whether implemented through funder requirements, institutional practices or controlled-access system, which collectively work to support responsible data sharing in the presence of high re-identification risks. To support the digital health research community, we developed a self-paced, online educational module for digital health researchers, trainees, and Institutional Review Board members (see: https://mhealthhub.org/portfolio/wristprint/). The module introduces learners to the dual imperatives of data sharing and privacy protection, illustrates risks through use case examples drawn from the creation of raw accelerometry data and provides practical guidance for preparing DMS plans. Because empirical evidence on the effectiveness of modality-specific educational strategies is limited, the module focuses on principles that may be generalizable to voice and other digital traces recognizing that certain data types may warrant tailored discussion as research evolves. By making this resource accessible and flexible, we aim to build consistent awareness and capacity across the ecosystem, ensuring that researchers can both maximize scientific value and protect participants.

Technologies used in digital health research will continue to expand in scope and application. Continuous monitoring multimodal wearables and AI-powered analytics hold extraordinary promises for advancing precision medicine and public health. As noted, opportunities come with risks that evolve more quickly than static policies can anticipate. If data once considered anonymous or de-identified are, in fact, at risk of re-identification, policies must adapt.

The NIH DMS Policy was a landmark step toward open science. Its success depends on our ability to align openness with protection of research participants, this involves a commitment to adapting guidance, strengthening consent and accountability and supporting the research community by creating the tools and processes to act responsibly. However, even strengthening informed consent has limitations. Researchers disclose risks known at the time of enrollment. Review boards are charged with assessing the probability and magnitude of that potential risk of harm. The consent communication can only convey what is known and, to some extent, the anticipated downstream re-identification harms that emerge as data moves through multi-party digital health infrastructures [23,24]. Compounding this limitation, the de-identification techniques that consent forms describe as potentially protective have been empirically shown to be insufficient, both in health records [25] and in genomic and large-scale digital datasets [14,25–27]. As Vayena and colleagues [28] have argued, data sharing must be pursued with both opportunity and risk in mind. The path forward is not to retreat from data sharing but to share more wisely. By anticipating risks and investing in education, we can safeguard participants and sustain the trust that underpins scientific discovery.
